# Efficient bio-production of citramalate using an engineered *Escherichia coli* strain

**DOI:** 10.1099/mic.0.000581

**Published:** 2017-12-12

**Authors:** Joseph P. Webb, S. Alison Arnold, Scott Baxter, Stephen J. Hall, Graham Eastham, Gill Stephens

**Affiliations:** ^1^​ Faculty of Engineering, University of Nottingham, Nottingham, UK; ^2^​ Ingenza Limited, Wallace Building, Roslin Biocentre, Edinburgh, UK; ^3^​ Lucite International, Wilton Centre, Wilton, Redcar, UK; ^†^​ Present address: Department of Molecular Biology and Biotechnology, University of Sheffield, Sheffield, UK.

**Keywords:** citramalic acid, methyl methacrylate, fed-batch fermentation, bio-based chemicals

## Abstract

Citramalic acid is a central intermediate in a combined biocatalytic and chemocatalytic route to produce bio-based methylmethacrylate, the monomer used to manufacture Perspex and other high performance materials. We developed an engineered *E. coli* strain and a fed-batch bioprocess to produce citramalate at concentrations in excess of 80 g l^−1^ in only 65 h. This exceptional efficiency was achieved by designing the production strain and the fermentation system to operate synergistically. Thus, a single gene encoding a mesophilic variant of citramalate synthase from *Methanococcus jannaschii,* CimA3.7, was expressed in *E. coli* to convert acetyl-CoA and pyruvate to citramalate, and the *ldhA* and *pflB* genes were deleted. By using a bioprocess with a continuous, growth-limiting feed of glucose, these simple interventions diverted substrate flux directly from central metabolism towards formation of citramalate, without problematic accumulation of acetate. Furthermore, the nutritional requirements of the production strain could be satisfied through the use of a mineral salts medium supplemented only with glucose (172 g l^−1^ in total) and 1.4 g l^−1^ yeast extract. Using this system, citramalate accumulated to 82±1.5 g l^−1^, with a productivity of 1.85 g l^−1^ h^−1^ and a conversion efficiency of 0.48 g_citramalate_ g^−1^_glucose_. The new bioprocess forms a practical first step for integrated bio- and chemocatalytic production of methylmethacrylate.

## Introduction

Methylmethacrylate (MMA) is used as the monomer to manufacture polymethylmethacrylate, a transparent, biocompatible material, better known as Perspex and Lucite. MMA is currently manufactured from petrochemical feedstocks, but is also an attractive target for more sustainable bio-production due to the favourable price (£1400/tonne [1]) and the large global market (2.1 million tonnes [2]). Despite numerous patent applications [3–5] on direct bio-production of methacrylic acid (MAA), a precursor for MMA, none of the proposed bioprocesses have yet been translated into a manufacturing process. The key problems hindering bio-production are (a) lack of enzymes to catalyse direct formation of MMA or MAA from bio-based feedstocks and (b) the toxicity of both compounds and their intracellular metabolites, due to their lipophilicity and reactivity with cellular components [6]. Similar problems with product toxicity are encountered commonly throughout the bio-economy [7–9] and solutions are needed. For these reasons, we investigated a combined bio- and chemocatalytic route, as an alternative to direct bio-production of MMA or MAA.

**Fig. 1.**
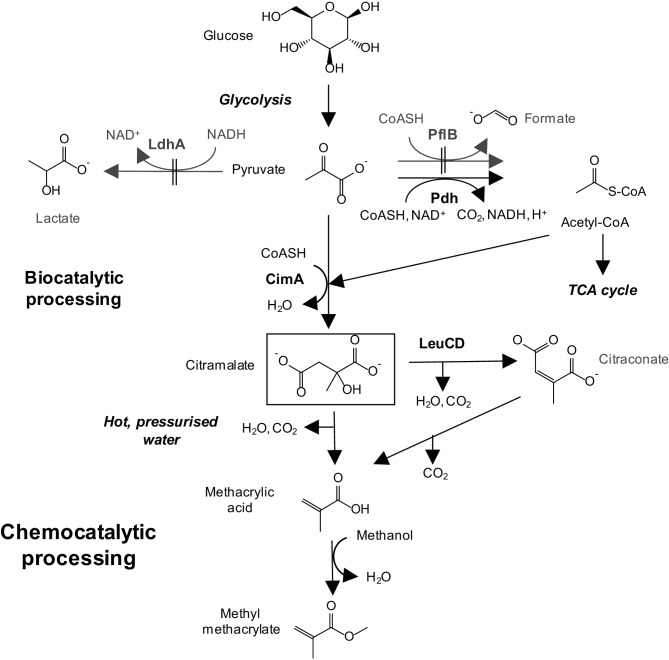
Citramalate production from glucose.

A facile, chemocatalytic route has already been developed to convert mixtures of naturally occurring, bio-derived di- and tricarboxylic acids to MAA in hot, pressurized water [10, 11]. Suitable acids include citric, itaconic, mesaconic, citraconic and citramalic acids [10, 11], and preliminary economic analyses, coupled with enzyme screening, indicated that citramalic acid was the most suitable target for bio-production (Lucite International, personal communication). Preliminary work also showed that citramalate was relatively non-toxic towards the host strain, since toxicity tests showed that the EC_50_ for citramalate towards *E. coli* was 173±7 mM (25.6±1 g l^−1^) compared with 13.2±1.7 mM (1.1±0.1 g l^−1^) for MAA (Table S1, available in the online version of this article). Therefore, we developed citramalic acid production as the initial biocatalytic step in the integrated bio- and chemocatalytic process. An *E. coli* strain was engineered to produce citramalate from glucose, by using citramalate synthase to catalyse the formation of citramalate from pyruvate and acetyl-CoA (Fig. 1) [12, 13], and a fed-batch bioprocess was also developed. Our patent describing this work was filed in 2014 and published in 2015 [14].

Since then, other workers have described a less efficient process, again using citramalate synthase, but also using multiple gene deletions (*gltA leuC ackA-pta poxB*) to control acetate formation [15, 16]. These interventions were required, because the fermentation strategy adopted (repeated fed-batch culture with excess glucose [15, 16]) is known to promote acetate formation by *E. coli* [17]. However, the use of multiple knockouts to control acetate and citraconate formation resulted in a double auxotroph, dependent upon addition of glutamate (5 g l^−1^) and leucine (1 g l^−1^) to obtain cell growth [15, 16]. In the repeated fed-batch system, a total of 110 g l^−1^ glucose, 25 g l^−1^ glutamate and 5 g l^−1^ leucine were needed to obtain 54.1 g l^−1^ citramalate, produced over 87 h, and acetate production (1.4 g l^−1^) was still observed, despite the deletions [16]. Unfortunately, however, the addition of glutamate and leucine almost doubles the feedstock costs compared with a process using glucose alone (based on current prices; Table S2). Although these expensive supplements could be replaced with peptone (15 g l^−1^), this strategy caused a decrease in product concentration to 46.5 g l^−1^ [15], and still increased the feedstock costs by 31 % compared with using glucose only (Table S2). Furthermore, the product stream contained both acetate and multiple organic contaminants from the peptone, thus adding to the costs of product recovery and effluent treatment. Therefore, we considered it timely to publish details of our patented production strain and fermentation process, since we obtained citramalate production to concentrations of >80 g l^−1^ in only 65 h [14]. Acetate was not produced, and a simple, low cost, mineral salts medium could be used, containing only glucose and 1.4 g l^−1^ yeast extract (<1 % of the feedstock cost).

## Methods

### Media and growth

Working stock cultures of *E. coli* strains were grown in LB, and stored as glycerol stocks (10 % w/v) at −80 °C. Where required, carbenicillin (50 µg ml^−1^) and/or chloramphenicol (34 µg ml^−1^) were added to media. All small-scale cultures in liquid media were grown at 37 °C, with orbital shaking at 250 r.p.m., in baffled shake flasks.

SM medium [1 l [18]] contained glycerol (30 ml; 500 g l^−1^), yeast extract (50 ml; 100 g l^−1^), MgSO_4_ (2 ml; 246.47 g l^−1^), salt solution (200 ml) containing Na_2_HPO_4_.12H_2_O (75.6 g l^−1^), KH_2_PO_4_ (15 g l^−1^), NH_4_Cl (5 g l^−1^), NaCl (2.5 g l^−1^), CaCl_2_ (55 mg l^−1^), and 100 µl of SM trace element solution containing FeSO_4_.7H_2_O (80 l^−1^ g), AlCl_3_.6H_2_O (10 g l^−1^), MnSO_4_.H_2_O (10 g l^−1^), CoCl_2_ (4 g l^−1^), ZnSO_4_.7H_2_O (2 g l^−1^), Na_2_MoO_4_.2H_2_O (2 g l^−1^), CuCl_2_.2H_2_O (1 g l^−1^) and H_3_BO_3_ (0.5 g l^−1^) dissolved in HCl (3 M). Glycerol, yeast extract, MgSO_4_.7H_2_O and salt solutions were prepared and autoclaved separately, and trace elements were sterilized by filtration using 0.2 µm, sterile filters. All solutions were mixed when cooled.

Fermentation medium (1 l) contained glucose (100 ml; 119 g l^−1^), MgSO_4_.7H_2_O (2 ml; 246.47 g l^−1^), polypropylene glycol (0.1 ml), salt solution (200 ml) containing K_2_HPO_4_ (73 g l^−1^), NaH_2_PO_4_.2H_2_O (18 g l^−1^), (NH_4_)_2_SO_4_ (10 g l^−1^), ammonium citrate (2.5 g l^−1^), and 2 ml of fermentation trace elements containing Na_2_EDTA.2H_2_O (22.3 g l^−1^), FeCl_3_ (10.03 g l^−1^), CaCl_2_.2H_2_O (0.5 g l^−1^), ZnSO_4_.7H_2_O (0.18 g l^−1^), CoCl_2_.6H_2_O (0.18 g l^−1^), CuSO_4_.5H_2_O (0.16 g l^−1^) and MnSO_4_.H_2_O (0.15 g l^−1^).

### Strain construction

Restriction digestions, transformations, SDS-PAGE and other standard molecular biological techniques, were performed using common procedures [19]. PCR was performed with KOD Hot Start Polymerase (Novagen) using standard protocols.

*E. coli* BW25113*ΔldhA *:: *cam^R^* was obtained from the Coli Genetic Stock Centre [20] and *pflB* was also deleted from the chromosome by λ red-mediated recombination [21] to produce *E. coli* BW25113*ΔldhAΔpflB*. The knockout resistance cassette was obtained by using pKD4 as the template DNA and the primers 5′-ACGCAGTAAATAAAAAATCCACTTAAGAAGGTAGGTGTTACGTGTAGGCTGGAGCTGCT TC-3′ and 5′-AGCCTTTATTGTACGCTTTTTACTGTACGATTTCAGTCAAATCTAAATGGGAATTAGCCATGGT CC-3′. The antibiotic resistance cassettes were cured by using the temperature sensitive plasmid pCP20 as described previously [21].

To produce pET20b(+)-*mjcimA3.7,* the *cimA3.7* gene [12], flanked by 5′ *NdeI* and 3′ *NotI* sites, was codon optimized for expression in *E. coli*, synthesized *de novo* (Biomatik) and ligated into pET20b(+) using standard techniques. The plasmid was transformed into *E. coli* BL21 (DE3)*plysS* to give the strain *E. coli* BL21 (DE3)*plysS* pET20b(+)-*mjcimA3.7*.

To produce pBAD24-*mjcimA3.7*, the *cimA3.7* gene was amplified from the cloning vector, pBMH-*mjcimA3.7*, using the primers 5′-AAAGCTAGCAGGAGGAATTCACCATGATGGTTCGTATCTTCG-3′ and 5′-TTTAAGCTTTACAGTTTACCAGTAACCTCACGC-3′ to introduce a 5′ *Nhe*I and 3′ *Hin*dIII restriction site. The gene was ligated into pBAD24 [22] and transformed into *E. coli* BW25113*ΔldhAΔpflB* to give the strain *E. coli* BW25113*ΔldhAΔpflB* pBAD24-*mjcimA3.7*, referred to as *E. coli* JW1.

### Citramalate production using harvested cells

Glycerol stocks of *E. coli* JW1 were used to inoculate SM glycerol medium (200 ml) to an OD_600nm_ 0.025. The culture was grown until the OD_600nm_ reached 0.6, and CimA3.7 expression was induced by the addition of *l*-arabinose (0.2 g l^−1^). Cells were harvested by centrifugation (4000 ***g***, 20 min, 4 °C) at an OD_600nm_ 3–4 and concentrated to a dry cell weight of 15 g l^−1^ in SM medium without NH_4_Cl or yeast extract, with glucose (20 g l^−1^) as the substrate. The cell suspensions (approximately 20 ml) were incubated in baffled flasks (500 ml) for 24 h at 250 r.p.m. and 37 °C.

### Citramalate synthase assay

Cell-free extracts of *E. coli* BL21 (DE3)*plysS* pET20b(+)-*mjcimA3.7* were prepared from cultures grown in LB medium (200 ml) 4 h after induction with IPTG (1 mM). Cells were harvested by centrifugation (4000 ***g***, 20 min, 4 °C) and resuspended in *N-*tris(hydroxyl)methyl-2-aminoethanesulfonic acid buffer (TES; 0.1 M, pH 7.5, 6 ml) containing MgCl_2_ (5 mM). Cells were lysed using a constant cell disrupter by two passages at 20 000 psi. CimA3.7 was partially purified by heating (60 °C, 10 min) and precipitated proteins were removed by centrifugation (12 000 ***g***, 10 min). The resulting cell-free extract was filtered (0.2 µm filter) and diluted to 1 mg ml^−1^ protein in TES buffer (0.1 M pH 7.5). CimA3.7 was assayed using a modified method [13]. The assay mixture (1 ml) contained: acetyl-CoA (1 mM), pyruvate (1 mM), partially purified CimA3.7 (1 mg ml^−1^; 200 µl) and TES buffer (0.1 M, pH 7.5) containing MgCl_2_ (5 mM). The reaction mixture was incubated at 37 °C and at regular intervals (10 min) samples (100 µl) were taken, mixed with 5,5′-dithio-*bis*-(2-nitrobenzoic acid) (0.56 mM), in Tris-HCl buffer (78 mM, pH 8; 900 µl), and the absorbance at 412 nm measured.

### Fed-batch fermentation

Inocula for fed-batch fermentations were developed by inoculating glycerol stocks (20 µl) of *E. coli* JW1 into fermentation medium (100 ml) and incubating overnight. The cultures were used to inoculate fermentation medium (3 l) in a Fermac 360 bioreactor (Electrolab; 5 l vessel). Cultures were grown at 37 °C, and the pH was maintained at pH 7.0 by automated addition of acid and base. The air flow rate was 3 l min^−1^ and the dO_2_ was maintained at 30 % of saturation by automatic control of the stirrer speed between 600–1200 r.p.m. A feed of glucose was started when the glucose had been consumed, as indicated by a sharp increase in dO_2_ and confirmed using glucose oxidase test strips. The flow rate was adjusted manually as required to maintain a pseudo-exponential growth rate of approximately 0.25 h^−1^ and avoid the accumulation of excess glucose in the culture, using the test strips as confirmation as above. CimA3.7 expression was induced by the addition of *l*-arabinose (0.2 g l^−1^). Feed regimes thereafter are described in the text.

### Analytical methods

Growth was monitored by measuring OD_600nm_. The samples were diluted in deionized water when the OD_600nm_ was >0.8. Dry cell weight was measured by centrifuging 1 ml samples in pre-weighed polypropylene tubes, removing the supernatant and drying the pellets at 80 °C to a constant weight. Samples were prepared for high-performance liquid chromatography (HPLC) analysis by boiling to inactivate the biocatalyst, centrifuging (12 000 ***g***, 5 min) and filtering the supernatants (0.45 µm filter). Samples (500 µl) were analysed using an Agilent 1200 series HPLC system with both UV (210 nm) and refractive index detectors, using a Rezex ROA Organic Acid H+column at 55 °C with 5 mM H_2_SO_4_ (0.5 ml min^−1^) as the mobile phase. Products were identified by comparing the retention times with authentic, commercial standards, and concentrations were determined from calibration curves.

## Results

### Design and construction of a citramalate-producing *E. coli* strain

We considered a number of options for citramalate production. Proof of concept for production of citramalate has already been obtained using itaconate [23] or citraconate [24] as the substrate for small-scale bioprocesses using harvested cells. However, the citraconate must be synthesized by the thermal decomposition of citric acid [10, 25, 26], which in turn needs to be produced by fermentation [27], increasing the cost. Similarly, itaconate is produced either by direct fermentation [28], or from citric acid as above [25, 26]. Furthermore, the growth and preparation of the harvested cells requires multiple unit operations, adding to process complexity and cost. Therefore, we designed a simpler, single-step fermentation of glucose directly to citramalate, using metabolically engineered *E. coli* cells [14]. The best characterized enzyme catalysing citramalate production is citramalate synthase (CimA; EC 2.3.1.182) from the thermophilic archeon, *Methanococcus jannaschii* [12, 13], which utilizes pyruvate and acetyl-CoA as the substrates. Since the latter metabolites are central metabolic intermediates, it should be possible to obtain citramalate production from glucose simply by expressing CimA in *E. coli* (Fig. 1 [14]). Since wild-type CimA is most active at high temperatures (e.g. 70 °C [13]), we expressed a mesophilic variant, CimA3.7, which was obtained by directed evolution [12]. The mutant enzyme contains five amino acid substitutions and is truncated at the C-terminal, resulting in higher activity at mesophilic temperatures and resistance to isoleucine feedback inhibition. This variant was used previously in multi-step, engineered pathways to produce 1-butanol and 1-propanol [12], indicating its suitability for bio-based chemical manufacturing.

Although CimA3.7 is active at 30 °C [12], our process was designed to operate at 37 °C, the optimum growth temperature for *E. coli*. Therefore, we tested the activity of CimA3.7 at this higher temperature. The *cimA3.7* gene was codon optimized for expression in *E. coli*, cloned into pET20b(+), and CimA activity was confirmed at 37 °C (Fig. S1). No activity was detected in control reactions lacking pyruvate or using cell-free extracts from a strain containing an empty pET20b(+) vector.

Expression of CimA3.7 from the T7 promoter of pET20b(+) resulted in a large proportion being expressed as insoluble protein (Fig. S2), which was unlikely to be active *in vivo*. To overcome this, the *cimA3.7* gene was cloned into pBAD24 [22] to produce pBAD24-*mjcimA3.7*, with CimA3.7 expression under the control of the arabinose-inducible, *araBAD* promoter, to provide better control of expression levels. Expression tests with varying *l*-arabinose concentrations (Fig. S3) showed that maximum production of soluble CimA3.7 was observed when cultures were induced with 0.2 g l^−1^ *l*-arabinose, with little formation of insoluble protein. Therefore, 0.2 g l^−1^ *l-*arabinose was used to induce CimA3.7 expression in subsequent experiments.

The production strain was then designed, based on the anticipated process configuration for industrial citramalate production. Although *E. coli* is an excellent host for heterologous expression of engineered metabolic pathways, and grows rapidly in minimal media, industrial fermentations are frequently plagued by unproductive conversion of glucose to acetic acid [17]. This decreases product yields and also causes inhibition of cell growth [29]. However, robust solutions to this problem are well known. By operating the fermentations as fed-batch cultures with a continuous, growth-limiting feed of glucose, acetate production can be avoided completely [30]. Therefore, we considered that any further metabolic engineering, beyond the overexpression of CimA3.7, could be minimized, and that there was no need to delete any of the genes that are known to be involved in acetate production. In addition to this, there was no need to delete either *leuC* or *leuD* [15], even though 3-isopropylmalate dehydratase is known to catalyse the conversion of citramalate to citraconate [12, 30–32]. Citraconate is a very good substrate for the hot, pressurized water process to produce MAA [10, 11], and, therefore, we considered that any formation of citraconate as a co-product alongside citramalate would be beneficial rather than detrimental (*vide infra*). However, industrial fed-batch cultures have to be operated at high biomass concentrations (>50 g l^−1^ dry weight), which can lead to oxygen-limited microenvironments, even within very well-mixed, high intensity fermentations [33]. Under oxygen limitation, *E. coli* switches to fermentative metabolism, with wasteful formation of fermentation products from pyruvate. Therefore, lactate dehydrogenase and pyruvate formate lyase were deleted to produce *E. coli* BW25113Δ*ldhA*Δ*pflB*, to prevent unproductive pyruvate metabolism [34], and thus maximize citramalate yields and simplify product recovery. *E. coli* BW25113Δ*ldhA*Δ*pflB* was then transformed with pBAD24-*mjcimA3.7* to produce *E. coli* JW1.

The production of citramalate from glucose was confirmed by using *E. coli* JW1 in small-scale biotransformations, and compared with wild-type *E. coli* BW25113 and *E. coli* BW25113Δ*ldhA,* both transformed with pBAD24-*mjcimA3.7,* to assess the impact of the deletions on aerobic growth and product formation (Table 1). Pre-induced, harvested cells were used in these tests to avoid induction of enzymes involved in acetate formation, and thus obtain an accurate comparison of product formation by the different strains.

**Table 1.** Citramalate production in small-scale biotransformations. Strains were grown and induced in SM medium (200 ml) with glycerol as the carbon source, to avoid glucose catabolite repression of the *araBAD* promoter. The cells were then harvested and resuspended to 15 g l^−1^ dry weight in fresh SM medium containing glucose (20 g l^−1^) but without yeast extract or NH_4_Cl. Results are an average of triplicate experiments and the error is the sd. nd, none detected.

Strain	Glucose consumed (g l^−1^)	Citramalate produced (g l^−1^)	Acetate produced (g l^−1^)
*E. coli* JW1	19.9±0.4	7.7±0.3	nd
*E. coli* BW25113 Δ*ldhA*Δ*pflB* pBAD24	17.9±0.3	nd	3.5±0.8
*E. coli* BW25113 Δ*ldhA* pBAD24-*mjcimA3.7*	18.1±0.2	7.4±0.5	nd
*E. coli* BW25113 pBAD24-*mjcimA3.7*	17.9±0.2	8.1±0.3	nd

After 24 h, the glucose had been consumed by *E. coli* JW1 and converted to citramalate (7.7±0.3 g l^−1^), a yield of 0.39±0.01 g_citramalate_ g^−1^_glucose_ (0.47±0.01 mol mol^−1^). No other organic acid contaminants (including acetate) were detected. This suggested that glucose was converted to citramalate and CO_2_ as the sole products. A control strain containing pBAD24 without *mjcimA3.7* also consumed the same amount of glucose but produced acetate as the sole metabolic product (3.5±0.8 g l^−1^). Therefore, *E. coli* JW1 could produce significant quantities of citramalate, and expression of CimA3.7 was sufficient to divert the C-flux away from acetate production and towards citramalate formation in the harvested, nitrogen-starved cells.

Wild-type *E. coli* BW25113 and *E. coli* BW25113 Δ*ldhA* (without the *pflB* knockout), both containing pBAD24-*mjcimA3.7,* produced similar concentrations and yields of citramalate as strain JW1, showing that neither of the knockouts had detrimental effects on product formation under aerobic biotransformation conditions in shake flasks.

### Development of a bioprocess for production of citramalate

**Fig. 2.**
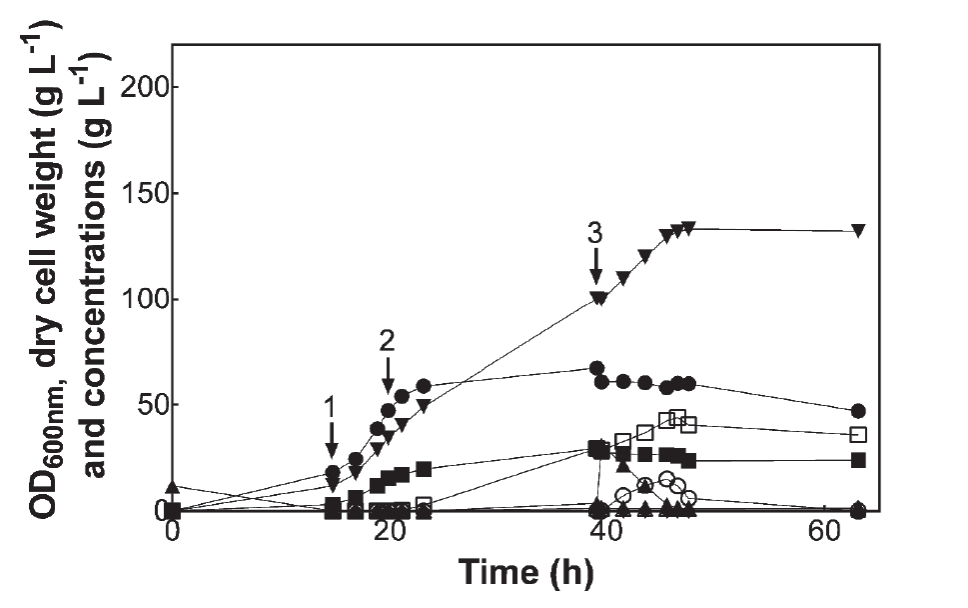
Fermentation for the production of citramalate. *E. coli* JW1 was grown with glucose (11.9 g l^−1^) until all the glucose had been consumed (point 1). A continuous, concentrated glucose feed was then started, with stepwise increases in the flowrate whilst maintaining glucose limitation. CimA3.7 expression was induced by adding *l*-arabinose (0.2 g l^−1^; point 2). The glucose feed was maintained for a further 19 h at a constant rate. The feed was then switched off (point 3) and a batch of glucose was added to the fermenter (120 g in total, 32 g l^−1^; 1 g l^−1^ gDCW^−1^ biomass). •–OD_600nm_, ■–dry cell weight, ▲–glucose measured, ▼–glucose consumed, ♦-acetate, ○–pyruvate, □–citramalate,–citraconate.

A fed-batch bioprocess was developed at 3 l scale, to investigate the scope for high productivity citramalate production. *E. coli* JW1 was grown initially with excess glucose (11.9 g l^−1^), until all the glucose had been consumed (Fig. 2; point 1). A concentrated glucose feed was then started, with stepwise increases in the flowrate to maintain both pseudo-exponential growth and glucose limitation, to avoid acetate accumulation and consequent growth inhibition [17, 35]. After 21 h, when the OD was 50, *l-*arabinose (0.2 g l^−1^) was added to induce synthesis of CimA3.7. Citramalate production was detected almost immediately after induction (Fig. 2; point 2), and continued at a rate of 1.6 g l^−1^ h^−1^, even though growth slowed and had almost stopped after 22 h. Despite this decrease in growth rate, glucose consumption continued, so that, after 39 h, a total of 365 g of glucose had been consumed and converted to 111 g of citramalate (29.5 g l^−1^), a yield of 0.30 g_citramalate_ g^−1^_glucose_ (0.37 mol mol^−1^). It should be noted that the culture medium contained only small quantities of yeast extract (only 0.85 g l^−1^). Since this represented <1 % of the total carbon-containing substrates added to the fermentation, the yeast extract had negligible effects on the calculated product yields.

Small quantities of citraconate (6 g; 1.61 g l^−1^) were also formed, presumably through dehydration of citramalate, catalysed by 3-isopropylmalate dehydratase [12, 30–32]. As noted above, this minor co-product can be tolerated since citraconate is converted to MAA alongside citramalic acid in the downstream chemical process [10, 11].

To test the potential of the non-growing cells to continue forming citramalate, the feed was stopped (Fig. 2; point 3), and a batch of glucose (120 g in total, 32 g l^−1^) was added to the bioreactor as a concentrated solution in water. The glucose was consumed within 8 h and converted to 25 g (7.5 g l^−1^) citramalate. Pyruvate (43 g; 11.25 g l^−1^) accumulated during the first 6 h after glucose addition, but the pyruvate was re-consumed once the glucose had been fully utilized. Surprisingly [17], acetate did not accumulate, despite the excess glucose present in the culture, presumably because any acetyl-CoA formed from glucose was consumed efficiently by the citramalate synthase reaction rather than being converted to acetate. There was no further increase in citraconate concentration and no further growth, indicating that the glucose was used only for citramalate production. After the glucose had been consumed, the concentration of citramalate began to fall, so there was no benefit in prolonged incubation.

Overall, the maximum titre of citramalate observed was 168 g (44 g l^−1^), produced from 509 g glucose in 63 h, including both the glucose used for cell growth and for citramalate production. Thus, the overall yield was 0.33 g_citramalate_ g^−1^_glucose_ (0.40 mol mol^−1^). During the production phase (i.e. after induction of CimA3.7), 383 g glucose was added to produce 168 g citramalate, and only 42 g biomass which indicates that 11 % of the total glucose added was used for biomass production and 44 % for citramalate production. The remaining carbon was lost as CO_2_ as a consequence of energy generation via the TCA cycle and aerobic respiration.

A further test of metabolic activity was made by harvesting a sample of cells after 63 h and resuspending the cells in SM biotransformation medium with 20 g l^−1^ glucose. After 24 h, all of the glucose (20.5±0.06 g l^−1^) had been consumed and 13.5±0.3 g l^−1^ citramalate produced, a yield of 0.66±0.02 g_citramalate_ g^−1^_glucose_ (0.8 mol mol^−1^). It should be noted that this apparently high yield value does not include the glucose utilized for cell growth, prior to harvesting from the culture, which must also be taken into account to give the overall yield across the whole bioprocess. A small amount of acetate (1.14±0.09 g l^−1^) was also produced.

**Fig. 3.**
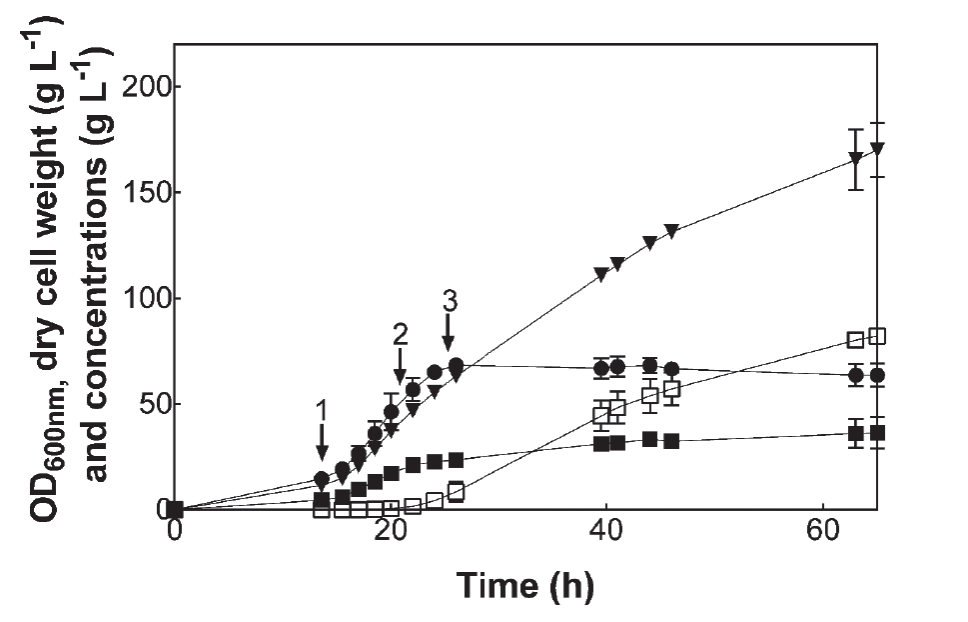
Citramalate production using a continuous glucose feed. Triplicate fermentations of *E. coli* JW1 were established as in Fig. 1, with the same glucose feeds to point 1 and 2. After induction with arabinose (point 2), the glucose feed rate was the same as Fig. 1 for the first 4 h. Thereafter (point 3), the feed rate was adjusted to deliver the correct ratio of glucose: cell dry weight to maintain a linear rate of product formation, based on rates measured in previous experiments. Residual glucose could not be detected. Data are the means of triplicate fermentations and the error bars indicate the sd. •–OD_600nm_, ■–dry cell weight, ▼–glucose consumed, □–citramalate.

Since the cells maintained metabolic activity even after the sustained period of starvation encountered at the end of this fermentation, the bioprocess was modified to provide a continuous glucose feed throughout the production phase. The initial stages of the bioprocess (Fig. 3) were operated in exactly the same way as the process shown in Fig. 2, with a continuous glucose feed started at point 1 and arabinose induction at point 2. The glucose feed was continued for 4 h after induction, to point 3, and then the feed rate was adjusted as required to maintain a linear rate of citramalate production. The required feed rate was estimated based on the previous glucose consumption and citramalate production rates (Fig. 2). The new bioprocess was repeated three times, and the mean data for these fermentations are reported (Fig. 3).

Once induced, citramalate production continued at a productivity of 1.85±0.02 g l^−1^ h^−1^, slightly higher than in the previous fed-batch fermentation (Fig. 2) and increased by 56 % compared with the addition of a single batch of excess glucose to stationary phase cells (Fig. 2). This shows that the use of a continuous, limiting glucose feed enhances citramalate production compared with a discontinuous fed-batch process, where the glucose is fed by adding batches of excess glucose.

The fermentations were terminated after 65 h, due to our limited capacity for shift work. Overall, 82±1.5 g l^−1^ of citramalate was produced from 172±11 g l^−1^ of glucose consumed, a yield of 0.48±0.03 g_citramalate_ g_glucose_^−1^ (0.58±0.03 mol mol^−1^). Therefore, the use of a continuous glucose feed had improved the overall titre and yield of citramalate by almost twofold compared with batch-wise glucose addition (Fig. 2). As before (Fig. 2), small quantities of citraconic acid (1.9±0.3 g l^−1^) were also produced, which further emphasizes that this is a minor co-product. Therefore, we conclude that it is not necessary to delete *leuC* and/or *leuD*.

The reproducibility of the process was excellent across the three replicate fermentations. Although the fermentations were terminated after 65 h for practical reasons, there is scope to continue the fermentations for longer periods to explore the possibility of obtaining higher titres of citramalate.

## Discussion

The reproducible, high titre production of citramalate (summarized in Table 2) was demonstrated using a metabolically engineered *E. coli* strain as the biocatalyst. The strain engineering was beguilingly simple, since expression of CimA3.7 coupled with only two knockouts was sufficient to divert acetyl-CoA and pyruvate flux efficiently towards citramalate production when *E. coli* JW1 was used in a fed-batch process with a continuous, limiting glucose feed. Thus, product titres of 82 g l^−1^ were obtained reproducibly over 65 h, with a productivity of 1.85 g l^−1^ h^−1^ and a conversion efficiency of 0.48 g_citramalate_ g^−1^_glucose._

**Table 2.** Comparison of fermentation processes for citramalate production

**Fermentation**	**Citramalate titre (g l^−1^)**	**Fermentation length (h)**	**Conversion efficiency**	
			**g g^−1^**	**mol mol^−1^**
Batch-wise glucose addition (Fig. 2)	44*	63	0.43	0.53
Continuous glucose feed (Fig. 3)	82±1.5	65	0.48±0.03	0.58±0.03
Wu and Eiteman [13]	46.5	132	0.63†	0.76†
Parimi *et al.* [14]	54.1	87	0.64†	0.78†

*Maximum titre detected.

†Product yields are over-estimates, since the conversion of glutamate, leucine or peptone to citramalate was not measured.

In part, this efficiency was due to the channelling of the central metabolic intermediates, pyruvate and acetyl-CoA, directly towards the formation of the naturally occurring intermediate, citramalate, in a single enzymatic step. Similar efficiencies are obtained in other bioprocesses where the chemical products are naturally occurring metabolites formed from central intermediates (e.g. the succinate, citrate, itaconate, poly-β-hydroxybutryrate and biobutanol fermentations) [36]. Our study indicates that there is considerable potential to develop this approach further, to produce a wider range of precursors for platform chemicals and monomers, or chiral starting materials for chemical synthesis.

Process efficiency was further enhanced by designing the production strain and the bioprocess to work synergistically together, so that acetate production could be avoided. This approach made it unnecessary to delete any of the genes involved in acetate production, so that there was no need to add large quantities of expensive nutritional supplements (glutamate or peptone) to overcome the auxotrophy created by deletion of *gltA* [15, 16]. Furthermore, citraconate was formed as a very minor co-product, and can be used as a substrate for MAA production in the hot water process, thus overcoming the need to delete *leuC*, and avoiding the need for costly leucine supplementation. Thus, our strain and process produced 50 % more citramalate in only 75 % of the time compared with previous processes [15, 16], and without formation of acetate as a co-product.

Although it appears that the citramalate yields (0.48 g_citramalate_ g^−1^_glucose_) are lower than in previous reports (0.64 g_citramalate_ g^−1^_glucose_) [15, 16], the latter studies involved the use of media supplemented either with substantial quantities of glutamate (25 g l^−1^) and leucine (5 g l^−1^) [16] or with peptone (15 g l^−1^) [15]. These nutrients can be incorporated directly into cell biomass, or used as energy sources, thus reducing the amount of glucose needed to sustain growth, and increasing the availability of glucose for citramalate production. The authors did not measure or account for the consumption of these additional nutrients, and calculated the yield of citramalate based on glucose consumption alone. Therefore, the apparent yields reported previously [15, 16] are likely to be substantial over-estimates. By contrast, the yields determined in our study are reasonable estimates of the true product yield on glucose, since the yeast extract supplement added to the cultures represented <1 % of the total carbon-containing substrates added to the cultures (i.e. the overall quantity of glucose fed was 172±11 g l^−1^ compared with 1.4 g l^−1^ yeast extract).

In conclusion, the patented process [14] for citramalate production provides the highest product titres reported to date, with excellent productivities. Product yields represent 58 % of the theoretical maximum and the process does not need expensive nutritional supplements to be added to the cultures. Therefore, the patented process represents a much more cost-effective system for citramalate production than the processes reported subsequently [15, 16], and provides a more attractive option for sustainable MAA manufacturing through a combined biological and chemical route. Such cost effectiveness is critical for bio-production of platform chemicals and monomers, due to the relatively low value of the end products.

The availability of the high titre, high yield bioprocess would also make citramalate an attractive bio-based precursor to manufacture other, previously inaccessible products with much higher values. Thus, citramalate has been used as a chiral synthon for preparation of chiral aliphatic sulphones [37], and for isoprenoid synthesis [38], but these processes have previously been limited by the high cost and limited availability of enantiopure citramalate. The further, deliberate conversion of citramalate to citraconic acid [12, 30–32, 39–41] may also have potential to provide a novel C5 building block/monomer. Therefore, the bioprocess for citramalate production has considerable future potential to manufacture methacrylate monomers and high value intermediates.

## Supplementary Data

576 Supplementary File 1
