# Supplementary material for: Efficient bio-production of citramalate using an engineered Escherichia coli strain
Source: Microbiology (Reading). 2017 Dec 12;164(2):133–41. doi: 10.1099/mic.0.000581 (PMC5882075; doi:10.1099/mic.0.000581)
Supplement: Supplementary File 1 [file mic-164-133-s001.pdf]

Supplementary material

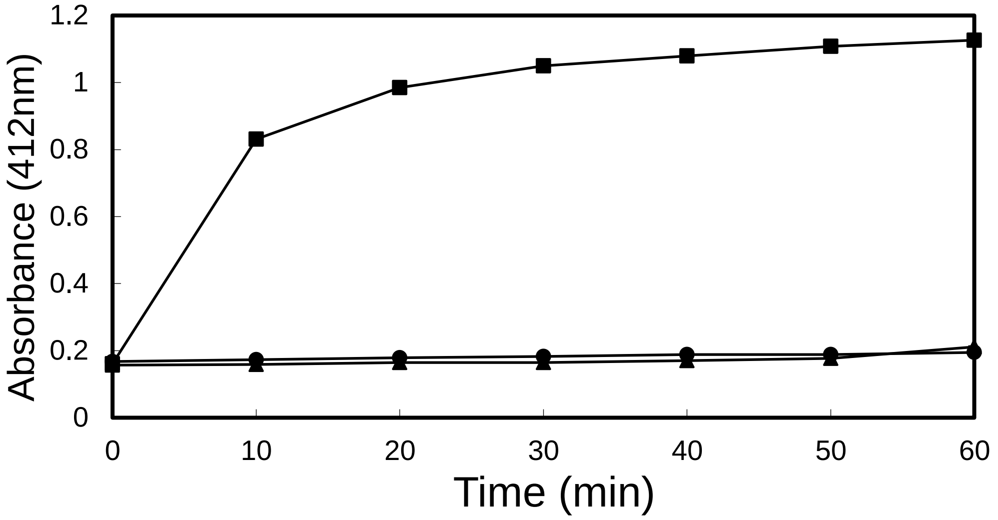

Figure S1: CimA3.7 activity assay

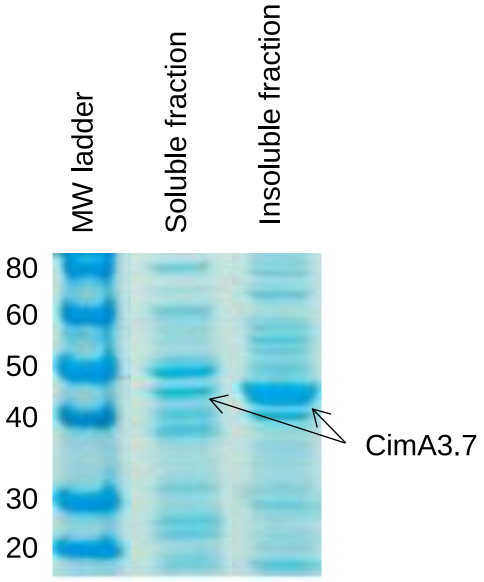

Figure S2: SDS-PAGE analysis of CimA3.7 expression with IPTG

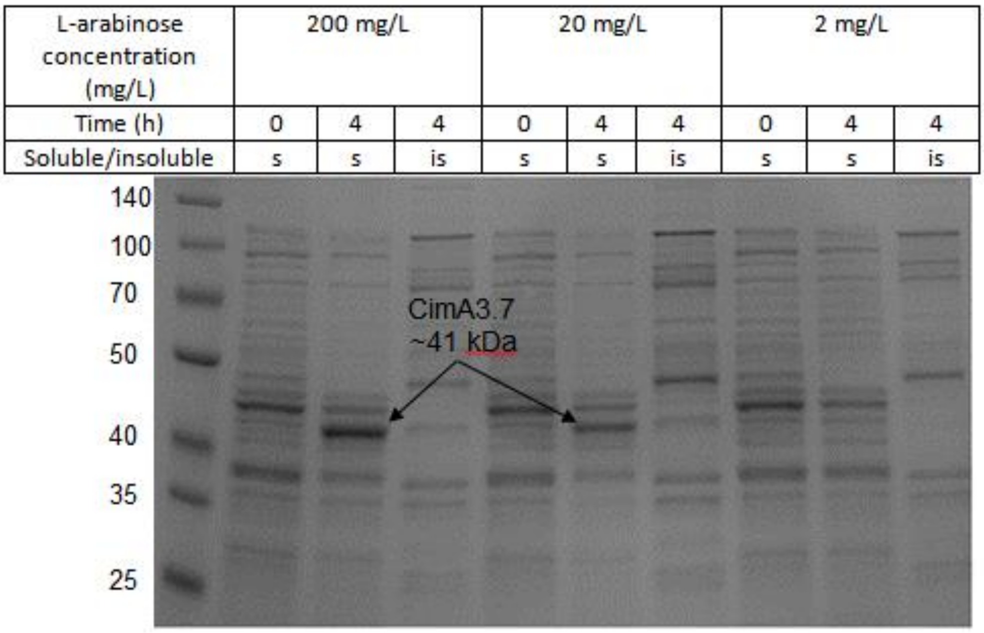

Figure S3: Optimisation of CimA3.7 expression from pBAD24

Table S1: EC_{50} of methacrylic and citramalic acid

	EC_{50} (mM)
**Methacrylic acid**	13.2 ± 1.7
**Citramalic acid**	173± 7

Table S2: Costing of fermentation media

**Organic nutrients, and the relevant reference.**	**Cost of nutrient (GBP/Kg)**	**Total added during fermentation (g/L)**	**Cost per litre of fermentation medium (GBP)**	**% cost of nutrient in comparison to cost of glucose added**
**Wu & Eiteman 2016 [13]**				
Glucose	21.4	85	1.819	
Peptone	48.6	15	0.729	40
**Parimi *et al.* [14]**				
Glucose	21.4	110	2.354	
Glutamate	50.6	25	1.265	53
Leucine	337.5	5	1.6875	71
**This Work**				
Glucose	21.4	172	3.612	
Yeast extract	32.5	1.4	0.0455	1.26

Supplementary References

Wahbi LP, Gokhale D, Minter S, Stephens GM. Construction and use of recombinant *Escherichia coli* strains for the synthesis of toluene *cis*-glycol. *Enzyme and Microbial Technology* 1996; 19:297–306.
